# Allergic Contact Dermatitis With Erythema Multiforme‐Like Features Induced by Isoconazole Nitrate

**DOI:** 10.1111/cod.70204

**Published:** 2026-06-12

**Authors:** Giorgia Sbarra, Giuseppe Demichele, Francesca Ambrogio, Alexandre Raphael Meduri, Paolo Antonio Danza, Caterina Foti

**Affiliations:** ^1^ Section of Dermatology and Venereology, Department of Precision and Regenerative Medicine and Ionian Area (DiMePRe‐J) University of Bari “Aldo Moro” Bari Italy; ^2^ Interdisciplinary Department of Medicine, Section of Occupational Medicine University “Aldo Moro” of Bari Bari Italy

**Keywords:** allergic contact dermatitis, case report, erythema multiforme‐like features, isoconazole nitrate, non‐eczematous contact dermatitis, patch testing

Polymorphous eruptions with erythema multiforme‐like features represent a rare non‐eczematous presentation of allergic contact dermatitis. Unlike true erythema multiforme, these reactions occur in the context of contact sensitization and may show targetoid, erythemato‐vesicular, urticarial or oedematous lesions, usually without mucosal involvement [1, 2].

## 1 Case Report

We report the case of a 22‐year‐old female student with a history of atopic dermatitis who developed an acute eruption after topical application of Travocort (isoconazole nitrate 1% + diflucortolone valerate 0.1%) for tinea corporis. The reaction started at the application site as an eczematous plaque and, over 14 days, progressed to a widespread polymorphous eruption.

Clinical examination showed a large erythematous‐oedematous plaque on the left lateral trunk with targetoid, urticarial and erythemato‐vesicular features, associated with similar lesions involving the inguinal folds and thighs. Direct mycological examination performed at presentation and after clinical resolution was negative.

**FIGURE 1:**
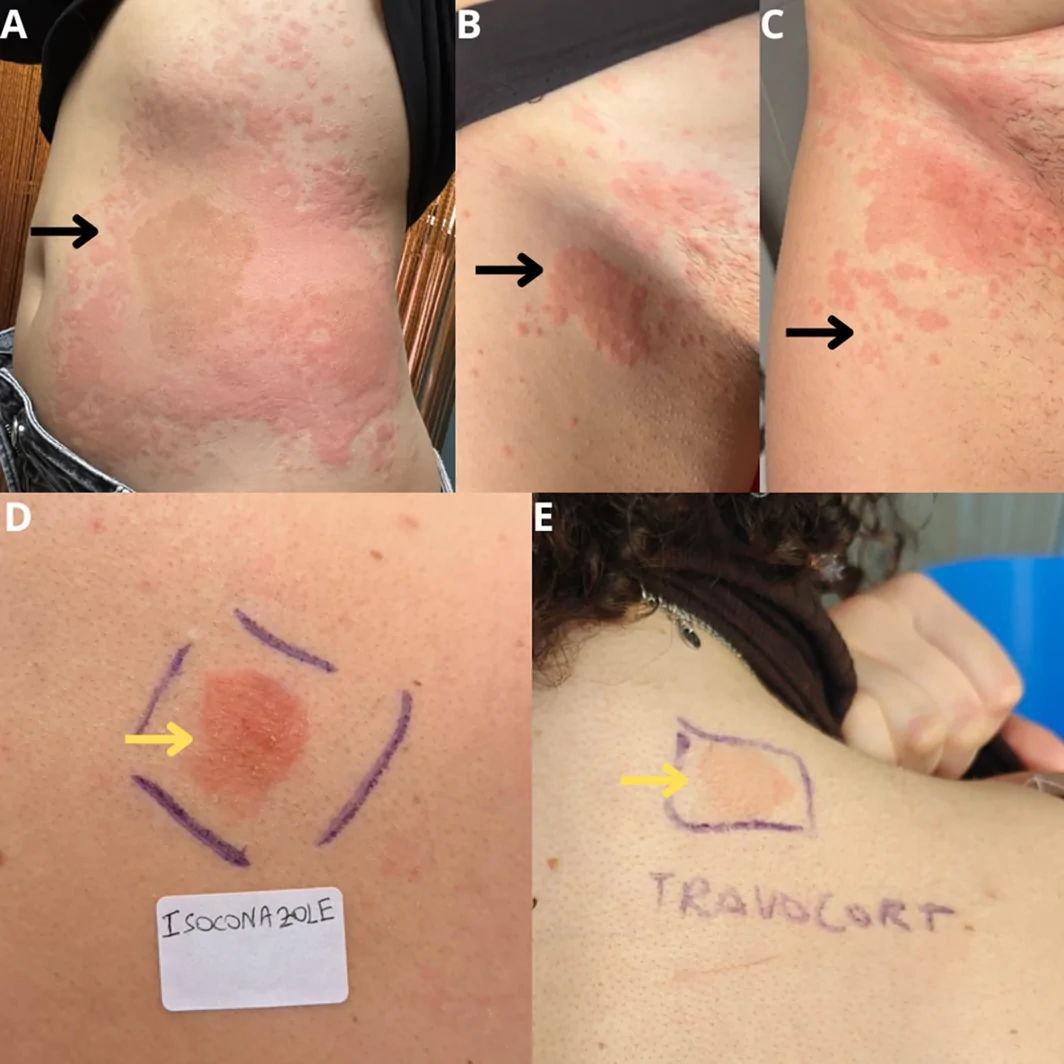
Clinical and patch test findings. (A) Large erythematous‐oedematous plaque on the left lateral trunk at the application site, with urticarial and erythemato‐vesicular features, indicated by the black arrow. (B, C) Similar polymorphous lesions involving the inguinal folds and thighs, indicated by black arrows. (D) Patch test reaction to isoconazole nitrate, showing a typical eczematous reaction, indicated by the yellow arrow. (E) Patch test reaction to Travocort cream, showing erythematous‐oedematous and urticarial morphology, indicated by the yellow arrow.

Patch tests were performed using the SIDAPA 2023 baseline series (Società Italiana di Dermatologia Allergologica Professionale e Ambientale; SmartPractice, Rome, Italy) in occlusion for 2 days (D2) with allergEAZE patch test chambers (SmartPractice, Phoenix, USA) on Soffix tape (Artsana, Grandate, Italy), and read on D2, D4 and D7 according to ICDRG criteria. Travocort cream was also tested (Figure 1).

The SIDAPA baseline series was negative at all readings. Travocort cream showed a positive reaction graded ++ at D2, D4 and D7, with erythematous‐oedematous and urticarial‐like morphology at D4, when photographs were taken. The active components were then tested separately: isoconazole nitrate, CAS 27523‐40‐6, 1% pet., and diflucortolone valerate, CAS 59198‐70‐8, 0.1% pet. Isoconazole nitrate showed a typical eczematous reaction graded ++ at D2, D4 and D7, whereas diflucortolone valerate remained negative at all readings.

The eruption progressively resolved after withdrawal of Travocort and treatment with topical clobetasol propionate.

## 2 Discussion

In the present case, we describe allergic contact dermatitis to isoconazole nitrate presenting with an unusual polymorphous clinical morphology.

This presentation required distinction from true erythema multiforme and dermatophytid reaction. True erythema multiforme was considered unlikely because mucosal involvement was absent and the eruption followed topical exposure. Dermatophytid reaction was also considered, given the eruption developed on presumably active tinea corporis; however, the onset at the treated site, rapid spreading after Travocort application, negative direct mycological examination at presentation and positive delayed patch test reactions were more consistent with allergic contact dermatitis.

Erythema multiforme‐like patterns in allergic contact dermatitis remain incompletely understood. Although their relationship with true erythema multiforme is unclear, both may share histological features such as upper dermal lymphoid infiltrate, exocytosis and keratinocyte necrosis, while mucosal involvement is typically absent in contact dermatitis. A type IV hypersensitivity mechanism mediated by sensitized T cells and cytokines, including interleukin‐2, interleukin‐1, interferon‐γ and tumour necrosis factor‐α, has been proposed; however, a type III immune‐complex mechanism has also been suggested. In a recent systematic review, medicaments were the most frequent allergen category and patch testing confirmed most reported allergens [2].

Isoconazole is an imidazole antifungal and a recognized, although uncommon, contact sensitizer, usually associated with classical eczematous dermatitis [3, 4]. However, few other atypical presentations have been described, such as erythematous and oedematous spreading lesions [4] and a pustular contact dermatitis, with subcorneal pustules and eosinophils [5]. Our case broadens this clinical spectrum by documenting a polymorphous, targetoid and urticarial‐oedematous presentation in confirmed isoconazole sensitization.

The urticarial‐like and erythematous‐oedematous morphology observed with Travocort may reflect modification of the reaction pattern by the corticosteroid component, as previously suggested for the same combination product [4] This discrepancy between the finished product and the isolated allergen highlights the importance of testing individual components when corticosteroid‐containing preparations produce atypical clinical or patch test morphology.

## Author Contributions

**Francesca Ambrogio:** conceptualization, writing – review and editing. **Giuseppe Demichele:** writing – review and editing, writing – original draft, data curation. **Paolo Antonio Danza:** writing – review and editing, writing – original draft. **Giorgia Sbarra:** conceptualization, writing – review and editing, data curation. **Caterina Foti:** conceptualization, methodology, writing – review and editing, supervision. **Alexandre Raphael Meduri:** writing – review and editing, writing – original draft.

## Consent

Written informed consent was obtained from the patient for the publication of the clinical images, in accordance with the principles of the Declaration of Helsinki.

## Conflicts of Interest

The authors declare no conflicts of interest.

## Data Availability

The data that support the findings of this study are available on request from the corresponding author. The data are not publicly available due to privacy or ethical restrictions.
